# Effect of combining inferior oblique muscle weakening procedures with exotropia surgery on the surgical correction of exotropia

**DOI:** 10.1371/journal.pone.0198002

**Published:** 2018-05-24

**Authors:** Seok Hyun Bae, Jisoo Kim, Ah Young Kim, Joo Yeon Lee, Mi Young Choi, Key Hwan Lim, Dong Gyu Choi

**Affiliations:** 1 Department of Ophthalmology, Kangnam Sacred Heart Hospital, Hallym University College of Medicine, Seoul, Korea; 2 Department of Ophthalmology, Chungbuk National University Hospital, Chungbuk National University College of Medicine, Cheongju, Korea; 3 Department of Ophthalmology, Ewha Womans University Mokdong Hospital, Ewha Womans University School of Medicine, Seoul, Korea; 4 Department of Ophthalmology, Hallym University Sacred Heart Hospital, Hallym University College of Medicine, Anyang, Korea; Faculty of Medicine, Cairo University, EGYPT

## Abstract

**Purpose:**

To determine whether the inferior oblique (IO) muscle weakening procedure combined with exotropia surgery affects the surgical correction of exotropia.

**Design:**

Institutional, retrospective study.

**Methods:**

We retrospectively reviewed the medical records of 310 patients who had undergone exotropia-correcting surgery combined with IO weakening (group A, 64 patients) or without IO weakening (group B, 246) with a postoperative follow-up of 6 months or more. The main outcome measures were the postoperative mean angle of horizontal deviation, the success rate, and the overcorrection rate. Surgical success was defined as an alignment between 10 prism diopters (PD) of exodeviation and 5 PD of esodeviation.

**Results:**

The postoperative mean angles of exodeviation, throughout the follow-up period, did not significantly differ between the groups. Although the surgical success rate was higher in group B at postoperative 1 month (p = 0.035), there was no statistical difference between the 2 groups from postoperative 6 months.: The final success rates were 56.3 and 51.6% (p = 0.509). The overcorrection rate was significantly higher in group A at postoperative 1, 6 and 24 months (p = 0.017, p = 0.028, p = 0.030, respectively); however, at the final follow-up, there was no overcorrection in either group.

**Conclusion:**

The overcorrection rate was higher in group A until postoperative 2 years, even though the mean angles of exodeviation and the success rates did not significantly differ between the 2 groups. Surgeons should be mindful of overcorrection when planning exotropia surgery combined with the IO weakening procedure.

## Introduction

In the primary position, the inferior oblique (IO) muscle causes excycloduction and elevation of the eye and also acts as an abductor [1–3]. Thus, theoretically, the IO weakening procedure can cause an esotropic shift in the primary position. Surgical weakening of the IO muscle can be performed, either unilaterally or bilaterally, to treat primary or secondary “inferior oblique overaction (IOOA)”. Wilson and Parks estimated the incidence of IOOA greater than 30% in those with acquired esotropia or intermittent exotropia [4]. When the surgery for the correction of exotropia is performed, the combined IO muscle weakening procedure is usually considered if IOOA ≥ +2 with/without V pattern exotropia [5]. A variety of IO weakening procedures, including IO recession, myotomy, myectomy, anterior transposition, denervation and muscle fixation, have been introduced [6–11]. There have been many previous studies on their effects on horizontal deviation. Minguini et al. reported that IO weakening did not affect the final results of horizontal rectus muscle surgery for correction of eso- or exotropia in the primary position [12]. However, Taylan et al. recorded a 4 PD (0–20) esoshift horizontally at distance and at near after IO weakening surgery. They stated, based on this result, that it was important to have a thorough knowledge of the possible effects on horizontal deviations when planning and performing strabismus surgery that aims to weaken the IO [13].

## Materials and methods

The medical records of 310 patients who had undergone surgery for exotropia with or without an IO weakening procedure and who had had at least 6 months of follow-up were retrospectively reviewed. Patients were excluded if they had a history of prior strabismus surgery, restrictive exotropia, paralytic strabismus, ocular disease other than strabismus, chromosomal anomalies, or systemic disorders such as congenital anomalies or neurologic disorders. The data collection included the age at surgery, sex, follow-up duration, pre- and postoperative deviation at distance and at near, cycloplegic refractive errors, amblyopia, associated strabismus, amount of IOOA, and stereopsis. This study adhered to the Declaration of Helsinki, and the protocol was approved by the Institutional Review Board of Hallym University Medical Center with an understanding on exemption from the informed consent for the study of retrospective collection of the clinical data. The data was accessed anonymously.

### Preoperative evaluation

All of the patients underwent complete ophthalmic examinations, including cycloplegic refraction with 1% cyclopentolate hydrochloride (Cyclogyl, Alcon Lab. Inc., Fort Worth, TX, USA) and 1% tropicamide (Mydriacyl, Alcon Lab. Inc.). The angle of deviation was determined by the alternate prism cover test at distance (6m) and at near (33 cm) using accommodative targets with the patients’ best optical correction. If the exodeviation at distance was larger than 10 PD or more comparable to that at near, we occluded one eye for 1 hour to eliminate fusional convergence, and then repeated the alternate prism cover test at distance and at near. The grading of IOOA were classified as +1 to +4 [14]. In maximal lateral version, a vertical deviation of approximately 10 degrees was grade +1, 20 degrees was +2, 30 degrees was +3, and 40 degrees was +4. Amblyopia was defined as a difference of 2 lines or more between monocular visual acuities. Vertical deviation was defined as hypertropia/hypotropia of 5 PD or more in the primary position. Sensory status was evaluated with the Titmus Stereotest (Stereo Optical Co., Inc., Chicago, IL, USA) and the Worth 4-Dot test at distance. Stereoacuity of 100 seconds of arc or better was defined as good stereopsis. Before the disruption of fusion by alternate prism cover test, the Worth-4-dot test was performed at distance (6m), and the results were recorded as follows: (1) fusion and (2) no fusion (of suppression and diplopia).

### Surgery

Four surgeons representing four institutions performed all of the surgeries under general anesthesia on the basis of the largest distance exodeviation measured. The surgical procedure, either bilateral lateral rectus (BLR) recession or unilateral lateral rectus recess-medical rectus resect (R&R), was selected by the surgeon according to his or her preference. Unilateral lateral rectus (ULR) recession could be performed in cases of exotropia less than 25 PD. The surgical dosages for distance exodeviation are presented in Table 1. Adjustable suture was not performed.

**Table 1:** Surgical dosages for exodeviation.

PD	BLR recession (mm)	R&R (mm)	ULR recession (mm)
**15**	4.0	4.0/3.0	8.0
**20**	5.0	5.0/4.0	9.0
**25**	6.0	6.0/4.5	10.0
**30**	7.0	6.5/5.0	
**35**	7.5	7.0/5.5	
**40**	8.0	7.5/6.0	
**50**	9.0	8.5/6.5	

PD = Prism diopters; BLR = Bilateral lateral rectus; R&R, Unilateral lateral rectus recess-medical rectus resect; ULR = Unilateral lateral rectus

Patients with +2 or more IOOA were candidates for the simultaneous IO weakening surgery. The type of IO weakening surgery, either recession or myectomy, was determined according to the surgeon’s preference. IO myotomy also was performed in some patients with +2 IOOA.

### Postoperative management

Postoperative alignment at distance and at near in the primary position was measured at postoperative 1, 6, 12, 24 months and at final follow-up. Patients with diplopia associated with postoperative esotropia were managed with alternating full-time patching until the diplopia disappeared. If the esodeviation persisted by postoperative 2 months, cycloplegic refraction was performed and refractive errors were recorrected. If the overcorrection persisted, base-out Fresnel press-on prisms (3M Press-On Optics; 3M Health Care, St Paul, Minnesota, USA) or prisms incorporated into regular spectacles were prescribed. Reoperation for undercorrection or recurrence was considered if the patient had exodeviation of 20 PD or more with poor control, and for overcorrection if 20 PD or more esodeviation persisted for more than 6 months postoperatively, or non-acceptance of conservative treatment by the patients even if esodeviation was less than 20 PD.

### Grouping

Exotropia-correcting horizontal muscle surgery combined with an IO weakening procedure was performed in 64 patients (group A), and horizontal muscle surgery only was performed in 246 (group B).

### Outcome measures

The main outcome measures included the mean angle of horizontal deviation, the success rate and the overcorrection rate on each follow-up. We also analyzed the effects of the type of IO weakening procedure and of the type of exotropia-correcting procedure on the surgical outcomes for exotropia in group A, respectively. Surgical success was defined as an alignment between 10 PD of exodeviation and 5 PD of esodeviation at distance on each follow-up. Overcorrection was defined as more than 5 PD of esodeviation at distance.

### Statistical analysis

Statistical analyses were performed with SPSS software version 23 (SPSS Inc., Chicago, IL). Continuous variables were expressed as mean ± standard deviations. The Mann-Whitney U-test and Pearson’s chi-square test were used to compare the preoperative characteristics between the groups. The Mann-Whitney U-test and Kruskal-Wallis test were used to compare the postoperative angle of deviation and follow-up duration, and the Pearson’s chi-square test was additionally employed to determine the inter-group success rates and overcorrection rates. P values less than 0.05 were considered statistically significant.

## Results

Of the 310 patients, the average age was 6.5 ± 3.5 years and the median age was 6.4 years. Table 2 provides a summary of the patients’ demographic characteristics. As will be noted, there were no significant inter-group differences in average age, sex ratio, refractive error, or the proportions of amblyopia and DVD. The postoperative follow-up duration was 32.3 months in group A and 31.1 months in group B (p = 0.591, Mann-Whitney U-test). The preoperative angle of exodeviation did not significantly differ: 26.0 ± 9.6 PD at distance and 25.9 ± 10.4 at near in group A and 26.0 ± 6.2 and 25.9 ± 8.5 in group B (p = 0.428 at distance, p = 0.801 at near, Mann-Whitney U-test). However, the proportion of patients with good stereopsis of 100 arcsec or better was 44.1% in group A and 81.1% in group B, and those with fusion on Worth-4 dot test, 24.0 and 57.6%, which differences were significant (p < 0.001 and p < 0.002, Pearson chi-square test). The patients in group A showed more vertical deviation (p < 0.001).

**Table 2:** Demographic data of patients in groups A and B.

Variables	Group A (n = 64)	Group B (n = 246)	p-value
**Age at surgery (years)**	6.7 ± 6.4	6.5 ± 2.2	P = 0.057*
**Sex (male/female)**	24/40	108/138	p = 0.356†
**Exodeviation (PD)**			
at distance	26.0 ± 9.6	26.0 ± 6.2	p = 0.428*
at near	25.9 ± 10.4	25.9 ± 8.5	P = 0.801*
**Spherical equivalent (D)**			
Dominant eye	0.2 ± 1.4	0.1 ± 1.4	p = 0.281*
Non-dominant eye	0.0 ± 1.9	-0.1 ± 1.6	p = 0.286*
**Amblyopia (%)**	27.0	14.9	P = 0.066†
**Associated strabismus**			
DVD (%)	7.8	2.7	P = 0.074†
Vertical deviation (%)	75.0	9.1	p<0.001†
**Good stereopsis in Titmus test (%)**	15/34 (44.1%)	167/206 (81.1%)	p<0.001†
**Fusion on Worth-4 dot test (%)**	6/25 (24.0%)	87/151 (57.6%)	P = 0.002†
**Postoperative follow-up duration (months)**	32.3 ± 28.9	31.1 ± 27.8	p = 0.916*

PD, prism diopters; D, diopters; DVD, dissociated vertical deviation

Good stereopsis = Stereoacuity of 100 seconds of arc or better

Group A: patients who underwent horizontal muscle surgery with IO weakening procedure

Group B: patients who underwent horizontal muscle surgery only

*Mann-Whitney U-test

†Pearson chi-square test

The procedures performed to weaken the IO in group A were IO myectomy in 36 patients (unilateral n = 17; bilateral n = 19), IO recession of 10 to 14 mm in 23 (unilateral n = 0; bilateral n = 23), and IO myotomy in 5 (unilateral n = 3; bilateral n = 2).

Tables 3 and 4 show the postoperative mean angles of exodeviation at distance and at near, respectively. From postoperative 1 month to final follow-up, there were no significant differences between the 2 groups.

**Table 3:** Postoperative angle of exodeviation (PD) at distance.

Postoperative	Group A (n = 64)	Group B (n = 246)	p-value*
1 month	2.8 ± 6.3	3.1 ± 5.0	0.489
6 months	4.4 ± 8.6	4.9 ± 6.8	0.926
12 months	4.3 ± 7.6	6.6 ± 7.8	0.146
24 months	7.6 ± 7.7	8.2 ± 8.3	0.962
Final follow-up	8.1 ± 8.2	10.1 ± 9.3	0.133

Group A = Patients who underwent horizontal muscle surgery with IO weakening procedure

Group B = Patients who underwent horizontal muscle surgery only

PD, prism diopters

*Mann-Whitney U test

**Table 4:** Postoperative angle of exodeviation (PD) at near.

Postoperative	Group A (n = 64)	Group B (n = 246)	p-value*
1 month	1.8 ± 6.5	3.2 ± 5.9	0.331
6 months	3.7 ± 8.2	4.7 ± 7.3	0.725
12 months	3.9 ± 6.8	6.2 ± 8.7	0.263
24 months	7.6 ± 8.0	8.1 ± 9.6	0.870
Final follow-up	7.4 ± 8.5	10.7 ± 10.8	0.056

Group A = Patients who underwent horizontal muscle surgery with IO weakening procedure

Group B = Patients who underwent horizontal muscle surgery only

PD, prism diopters

*Mann-Whitney U test

Surgical success was achieved for 69.8% of patients in group A and 81.9% in group B at postoperative 1 month, which showed better successful outcome in group B (p = 0.035, Pearson chi-square test). However, there were no significant differences in the success rates at postoperative 6, 12, or 24 months (p = 0.250, p = 0.472, p = 0.425, respectively) (Table 5). Indeed, the final success rates were very similar (group A = 56.3%, group B = 51.6%; p = 0.509) for mean follow-up periods of 32.3 ± 28.9 and 31.1 ± 27.8 months, respectively.

**Table 5:** Surgical success rates for horizontal alignments (%) in groups A and B.

Postoperative	Group A (n = 64)	Group B (n = 246)	p-value*
1 month	69.8	81.9	0.035
6 months	65.5	73.3	0.250
12 months	64.4	58.6	0.472
24 months	46.7	54.7	0.425
Final follow-up	56.3	51.6	0.509

Surgical Success: 5 PD esodeviation ~ 10 PD exodeviation

Group A: patients who underwent horizontal muscle surgery with IO weakening procedure

Group B: patients who underwent horizontal muscle surgery only

*Pearson chi-square test

On the other hand, it should be noted that the overcorrection rate was significantly higher in group A until 2 years after surgery (p < 0.05, Pearson chi-square test), with the exception of 1 year postoperatively (p = 0.060, Pearson chi-square test); nonetheless, no patient in either group was overcorrected at final follow-up (Table 6). Among group A, there was no statistically significant overcorrection-rate difference according to the type of IO weakening procedure (p > 0.05, Pearson chi-square test) (Table 7).

**Table 6:** Overcorrection rates (%) in groups A and B.

Postoperative	Group A (n = 64)	Group B (n = 246)	p-value*
1 month	9.5	2.9	0.022
6 months	9.1	2.3	0.017
12 months	8.9	2.8	0.060
24 months	6.7	0.7	0.025
Final follow-up	0	0	-

Overcorrection > 5 PD esodeviation

Group A: patients who underwent horizontal muscle surgery with IO weakening procedure

Group B: patients who underwent horizontal muscle surgery only

*Pearson chi-square test

**Table 7:** Overcorrection rates (%) according to type of IO weakening procedure.

Postoperative	IO myectomy (n = 36)	IO recession (n = 23)	IO myotomy (n = 5)	p-value*
1 month	5.6	18.2	0	0.212
6 months	7.1	13.6	0	0.555
12 months	8.0	12.5	0	0.715
24 months	11.1	0	0	0.490
Final follow-up	0	0	0	-

IO, inferior oblique

Overcorrection > 5 PD esodeviation

*Pearson chi-square test

Table 8 shows the pre- and postoperative mean angles of exodeviation according to the surgical methods for correction of exotropia in group A. The mean angles of exodeviation did not show any statistical difference from postoperative 1 month to final follow-up (p > 0.05, Kruskal-Wallis test). Neither surgical success nor overcorrection rate was significantly different according to the surgical method for correction of exodeviation (p > 0.05, Pearson chi-square test, Table 9)

**Table 8:** Pre- and postoperative angles of exodeviation (PD) according to surgical methods for correction of exodeviation in group A.

	BLR recession	R&R	ULR recession	p-value*
**Preoperative (PD)**	29.1 ± 9.1	31.3 ± 9.2	17.2 ± 3.5	<0.001
**Postoperative (PD)**				
1 month	3.1 ± 6.5	1.0 ± 4.5	2.8 ± 6.5	0.708
6 months	3.9 ± 9.1	4.8 ± 5.8	5.5 ± 8.4	0.986
12 months	4.6 ± 6.5	8.6 ± 8.2	1.4 ± 8.7	0.253
Final follow-up	9.0 ± 8.4	9.5 ± 10.3	5.7 ± 6.7	0.371
**Follow-up duration (mo)**	28.9 ± 20.7	31.6 ± 27.1	39.8 ± 42.1	0.971

Group A = Patients who underwent horizontal muscle surgery with IO weakening procedure

PD, prism diopters

*Kruskal-Wallis test

**Table 9:** Surgical success and overcorrection rates according to surgical methods for correction of exodeviation in group A.

Surgical outcomes	BLR recession	R&R	ULR recession	p-value*
**Success rate (%)**				
Postoperative 1 month	64.9	75.0	77.8	0.584
Postoperative 6 months	63.9	80.0	64.3	0.773
Postoperative 12 months	64.0	71.4	61.5	0.905
Final follow-up	52.6	50.0	66.7	0.786
**Overcorrection rate (%)**				
Postoperative 1 month	10.8	12.5	5.6	0.786
Postoperative 6 months	11.1	0	7.1	0.690
Postoperative 12 months	8.0	0	15.4	0.500
Final follow-up	0	0	0	-

Surgical Success: 5 PD esodeviation ~ 10 PD exodeviation

Overcorrection > 5 PD esodeviation

Group A = Patients who underwent horizontal muscle surgery with IO weakening procedure

BLR = Bilateral lateral rectus; R&R, Unilateral lateral rectus recess-medical rectus resect; ULR = Unilateral lateral rectus

*Pearson chi-square test

Table 10 shows the outcomes of exotropia surgery combined with the unilateral or bilateral IO weakening procedure in group A at postoperative 2 years. The mean angles of exodeviation at distance, with the two procedures, were 6.4 ± 8.5 PD and 8.1 ± 7.4, respectively (p = 0.579, Mann-Whitney U-test), and at near, 6.7 ± 9.8 and 8.1 ± 7.0 (p = 0.592), respectively, at postoperative 2 years. The 2-year success rates of the unilateral and bilateral IO weakening procedures were 50.0 and 45.0%, respectively, which were not significantly different (p = 0.796, Pearson chi-square test). The overcorrection rate also showed no significant difference (10.0 and 5.0%, respectively; p = 0.605).

**Table 10:** Outcomes of exotropia surgery combined with unilateral or bilateral IO weakening procedure in group A (n = 64) at postoperative 2 years.

Surgical outcomes	Unilateral IO weakening (n = 20)	Bilateral IO weakening (n = 44)	p-value
Mean angle of exodeviation (PD) at distance	6.4 ± 8.5	8.1 ± 7.4	0.579*
Mean angle of exodeviation (PD) at near	6.7 ± 9.8	8.1 ± 7.0	0.592*
Success rate (%)	50.0	45.0	0.796†
Overcorrection rate (%)	10.0	5.0	0.605†

IO, inferior oblique; PD, prism diopters

Surgical Success: 5 PD esodeviation ~ 10 PD exodeviation

Overcorrection > 5 PD esodeviation

*Mann-Whitney U-test

†Pearson chi-square test

Reoperations for recurrent exotropia were performed on 6 patients (9.4%) in group A and 40 (16.3%) in group B (p = 0.167, Pearson chi-square test). None underwent reoperation for consecutive esotropia.

## Discussion

Horizontal rectus muscle surgery combined with the IO weakening procedure might be necessary for correction of exotropia associated with excessive elevation in adduction. In the primary position, IO contraction causes not only excycloduction and elevation but also abduction of the eyeball. In light of this fact, the IO weakening procedure potentially causes an esoshift in the primary position [1–3]. Urist observed that bilateral recession of the IO muscles had but little effect on horizontal deviation [15]. Guzzinati published a report on a series of 20 cases, which showed varying influences on horizontal deviation after IO weakening procedures but no statistically significant change between the pre- and postoperative periods [16]. Taylan et al. reported a median 4 PD (range: 0–20 PD) of esoshift horizontally at distance and at near in the primary position, regardless of the type of surgery for IO [13]. Stager et al. reported that there was no significant horizontal change in 84% of their patients, and that in those who did manifest a change, the amount was never greater than 8 PD [17]. Minguini et al. concluded that IO weakening combined with horizontal muscle surgery did not affect the final outcome of horizontal surgery in the primary position [12].

In the present study, the mean angles of exodeviation did not statistically differ between the 2 groups throughout the postoperative follow-up period. And even though the success rate in group A was significantly lower at postoperative 1 month, there was no inter-group difference from postoperative 6 months to final follow-up. However, the overcorrection rates were higher in group A than in group B until 2 years, though neither group showed any overcorrection at the final follow-up. Most of the patients in whom surgery was unsuccessful were undercorrected; the proportion of overcorrection cases was relatively small (less than 10%, even in group A). So, our results suggest that although IO weakening surgery did not significantly influence the overall success rates and mean exodeviation angles after exotropia surgery, surgeons should keep the possibility of overcorrection in mind when planning and performing intermittent exotropia surgery combined with the IO weakening procedure.

The present study has some limitations. First, it was a retrospective study, and the surgeon determined the method of exotropia surgery according to his or her preference. Thus, minor selection bias could have occurred. Second, the surgeries were performed by four surgeons in our study, which fact might cause heterogeneous results, even if they used the same surgical table for the correction of exodeviation (Table 1). From another perspective, however, the surgical bias potential to this situation was minimized. Third, we did not analyze the results according to the amount of horizontal rectus muscle surgery. Therefore, there is a possibility that the amount of horizontal rectus muscle surgery affected the results. Fourth, the preoperative sensory status, as indicated by good stereopsis of 100 arcsec or better or fusion on Worth-4 dot test, was significantly better in group B than in group A, which might have affected the surgical results. Choi et al. reported that the rate of DVD or IOOA in primary infantile exotropia was higher than in early-onset intermittent exotropia [18]. Thus, patients with primary infantile exotropia might have been included, especially in group A, which would explain the sensory-status difference between the 2 groups. Therefore, prospective long-term-follow-up studies on a larger number of patients are needed.

In conclusion, we found no significant differences in the rate of surgical success between patients with an exotropia-surgery + IO weakening procedure and those with exotropia surgery only. However, a tendency toward overcorrection in patients who had undergone horizontal muscle surgery combined with an IO weakening procedure was observed. Our study suggests that strabismus surgeons need to be aware of overcorrection when planning and performing exodeviation surgery combined with an IO weakening procedure, though further study is needed.

## Supporting information

S1 File (XLSX)
